# Gata3 targets Runx1 in the embryonic haematopoietic stem cell niche

**DOI:** 10.1002/iub.2184

**Published:** 2019-10-21

**Authors:** Simon R. Fitch, Chrysa Kapeni, Aikaterini Tsitsopoulou, Nicola K. Wilson, Berthold Göttgens, Marella F. de Bruijn, Katrin Ottersbach

**Affiliations:** ^1^ MRC Centre for Regenerative Medicine University of Edinburgh Edinburgh UK; ^2^ Cambridge Institute for Medical Research and Cambridge Stem Cell Institute University of Cambridge Cambridge UK; ^3^ MRC Molecular Haematology Unit, MRC Weatherall Institute of Molecular Medicine University of Oxford Oxford UK

**Keywords:** aorta‐gonads‐mesonephros, Gata3, haematopoietic stem cells, niche, Runx1, stromal cells

## Abstract

Runx1 is an important haematopoietic transcription factor as stressed by its involvement in a number of haematological malignancies. Furthermore, it is a key regulator of the emergence of the first haematopoietic stem cells (HSCs) during development. The transcription factor Gata3 has also been linked to haematological disease and was shown to promote HSC production in the embryo by inducing the secretion of important niche factors. Both proteins are expressed in several different cell types within the aorta‐gonads‐mesonephros (AGM) region, in which the first HSCs are generated; however, a direct interaction between these two key transcription factors in the context of embryonic HSC production has not formally been demonstrated. In this current study, we have detected co‐localisation of Runx1 and Gata3 in rare sub‐aortic mesenchymal cells in the AGM. Furthermore, the expression of *Runx1* is reduced in *Gata3* ^*−/−*^ embryos, which also display a shift in HSC emergence. Using an AGM‐derived cell line as a model for the stromal microenvironment in the AGM and performing ChIP‐Seq and ChIP‐on‐chip experiments, we demonstrate that *Runx1*, together with other key niche factors, is a direct target gene of Gata3. In addition, we can pinpoint Gata3 binding to the *Runx1* locus at specific enhancer elements which are active in the microenvironment. These results reveal a direct interaction between Gata3 and Runx1 in the niche that supports embryonic HSCs and highlight a dual role for Runx1 in driving the transdifferentiation of haemogenic endothelial cells into HSCs as well as in the stromal cells that support this process.

**Abbreviations**
Ack9: acetylated histone H3 Lysine 9
AGM: aorta‐gonads‐mesonephros
Ao: dorsal aorta
ChIP: chromatin immunoprecipitation
DHS: DNase I hypersensitive site
E: embryonic day
HSC: haematopoietic stem cell
IAHC: intra‐aortic haematopoietic cluster
ITD: internal tandem duplication
PSC: pluripotent stem cell
SNS: sympathetic nervous system

## 1 INTRODUCTION

Haematopoietic stem cells (HSCs) are clinically highly relevant cells that have been widely used for cell replacement therapies; however, their use for transplants is limited by the availability of compatible donors. Considerable effort is therefore being invested into the development of protocols for the derivation of HSCs from pluripotent stem cells (PSCs) in vitro as these could be derived from the patient's own cells. PSC differentiation into transplantable HSCs has proven to be challenging, although significant progress has been made in recent years.^1^, ^2^ One important component for success in these endeavours is a detailed understanding of how the embryo generates HSCs during development in vivo. The first and most robust production of HSCs occurs in the aorta‐gonads‐mesonephros (AGM) region starting from E10.5.^3^, ^4^, ^5^ The generation of HSCs involves the transdifferentiation of specialised haemogenic endothelial cells into blood cells in a process termed endothelial‐to‐haematopoietic transition, which is regulated by key transcription factors such as Runx1, Gata2, and Gfi1 and major signalling pathways, such as Notch and Bmp, which interact in a finely‐tuned manner (recently reviewed in^6^).

This process is also embedded in and regulated by the local microenvironment, which in the AGM appears to be especially supportive to the generation of HSCs (reviewed in^7^). The AGM haematopoietic niche is very dynamic and includes contributions from tissues that develop in its vicinity at the time of HSC emergence, such as the sympathetic nervous system (SNS)^8^ and the gut.^9^ Furthermore, the AGM was demonstrated to contain mesenchymal stem/stromal cell activity,^10^ and the ventral sub‐aortic mesenchyme is a rich source of many haematopoiesis‐regulatory factors.^11^, ^12^, ^13^, ^14^ This mesenchymal population appears to be very heterogeneous, and very little is currently known about its developmental origins, whether it is maintained by a local stem cell population and where the key HSC niche cells are located.

We demonstrate here that two important haematopoietic transcription factors, Gata3 and Runx1, both of which also perform important roles in adult haematopoietic lineages,^15^, ^16^ are co‐expressed in the sub‐aortic mesenchyme of the AGM and that their interaction may regulate the timing of HSC emergence. Furthermore, using AGM‐derived stromal cell lines as a model,^11^, ^13^, ^17^ we demonstrate that *Runx1* as well as other important haematopoietic factors such as *Kit* and *Cxcl12* are direct targets of Gata3. This highlights that some key HSC regulators such as Runx1 can perform cell‐intrinsic roles and at the same time function within the HSC niche.

## 2 EXPERIMENTAL PROCEDURES

### 2.1 Mice

Wild‐type C57BL/6, *Gata3* knockout,^18^ *Gata3‐LacZ* knockin,^19^ *Gata3‐GFP* knockin,^20^ or *Runx1‐LacZ* knockin^21^ mice were mated for embryo generation. The day of plug detection is designated as day 0. All mice were housed according to institute regulations, and procedures were carried out in compliance with UK Home Office licenses.

### 2.2 Long‐term transplantations

Dissected AGMs were dissociated with 0.125% collagenase and single cell suspensions injected into irradiated (split dose of 9.5 Gy γ‐irradiation) recipient mice together with 200,000 spleen helper cells. Donor contribution was measured at 1 and 4 months post‐transplantation by flow cytometry, using antibodies specific to the CD45.1 and CD45.2 isoforms. Mice were considered positive for repopulation if donor chimerism exceeded 5%.

### 2.3 Immunohistochemistry, immunocytochemistry and X‐gal staining

Embryos were fixed with 2% paraformaldehyde at 4°C, equilibrated overnight at 4°C in 30% sucrose and then snap‐frozen in Tissue Tek (Sakura Finetek). Ten micrometre cryosections were prepared and stained with anti‐GFP (chicken; Life Technologies), anti‐Runx1 (rabbit; Abcam), anti‐CD34 (FITC), anti‐chicken‐Alexa647 (Millipore), anti‐rabbit‐Alexa555, anti‐Kit (goat; R&D Systems), and anti‐goat‐Alexa488 before mounting in DAPI‐containing Vectashield (Vectorlabs). UG26‐1B6 cells grown on microscope slides were stained with anti‐Gata3 (rat; Absea), anti‐rat‐biotin (BD Biosciences), and Streptavidin‐Cy5 (Jackson Immunoresearch) before mounting in DAPI‐containing Vectashield. X‐gal staining of *Gata3* ^*+/lz*^ and *Runx1* ^*+/lz*^ embryos was carried out as described previously.^22^ Cryosections were prepared and counterstained with Neutral Red. Brightfield images were acquired with a Zeiss AxioSkop2 Wide‐Field Microscope and fluorescent images with a Widefield Zeiss Observer and analysed using Zen software.

### 2.4 Gene expression

Tissues and cells were dissociated in Trizol (Life Technologies) and RNA isolated and DNAse‐treated according to manufacturer's instructions. First strand cDNA was generated with Superscript II (Invitrogen) and conventional RT‐PCR performed with primers for *Gata3* (forward: CGAAACCGGAAGATGTCTAGC; reverse: AGGAACTCTTCGCACACTTGG), *Runx1* (forward: CGGAGGGAAACTGTGAATGC; reverse: CCCAAAGCTGTAGCTGTCTC), *Cxcl12* (forward: TTTCACTCTCGGTCCACCTC, reverse: TAATTTCGGGTCAATGCACA), and *Actb* (forward: CCTGAACCCTAAGGCCAACCG, reverse: GCTCATAGCTCTTCTCCAGGG).

### 2.5 Cell culture

The UG26‐1B6 and KH23 stromal cell lines were grown at 33°C in medium containing 50% Myelocult M5300 (Stem Cell Technologies), 35% α‐MEM (Invitrogen), 15% fetal calf serum (Sigma Aldrich), 0.5% penicillin–streptomycin (Sigma Aldrich), and 10 μM β‐mercaptoethanol.

### 2.6 ChIP‐qPCR and ChIP‐Seq

Chromatin immunoprecipitation (ChIP) was performed using an anti‐Gata3 antibody (rat, Absea or goat, Santa Cruz), an anti‐H3K9Ac antibody (rabbit, Millipore) and their respective IgG controls. ChIP material was analysed by qPCR using the following primers: *Mafk* (forward: CTCGTGTTGTCTTTCCGCAC, reverse: CGACCTTGGGATGTAGCCAA); *Tcf3* (forward: GGAACTATCTTCCTACGCGGC, reverse: TAATGGTCCTTCCCGCTTGC); *Ccdc34* (forward: TGATGTCAGCACTCTGCCTC, reverse: TTGTGCCAGGACAAGCAGAT); *Cd28* (forward: TCTTTCCATTGCTTTGCGGC, reverse: TGGCCACTCACTATGCATCT); *Klf6* (forward: ATCTGCAGCTGCTGGACAAC, reverse: TGCATGAGTCAGCGTCTTCA); *Lmo7* (forward: GGTGGCAGTCTTGGAAGTCA, reverse: TGAGTAACCAGCGACACACC); *Cxcl12* (forward: ATGTGCGCCCTGCAGATATT, reverse: AGCGTGAGTCATCGACTTGG); *Runx1* (forward: AGCAGCACCGAATGAGTCAA, reverse: CGTATGCTGGGCCTTTCCTC); *Runx1 + 23* (forward: CGAAAAATAAACCGGCAGTTGA, reverse: CAAGCTGCCCACGTTATCAGT); *Runx1 + 110* (forward: CCTTTACATCTCCTCAATCATTGCT, reverse: TCCAAATGCCCCCAGTCA); *Runx1 + 3* (forward: ACCACAGCCTGCCACTCCTA, reverse: AGGGAGCACAGGCCATAAATTA); *Gapdh* (forwards: CAAGGCTGTGGGCAAGGT, reverse: TCACCACCTTCTTGATGTCATCA). ChIP‐Seq experiments were carried out as described previously.^23^ Samples were amplified, sequenced on an Illumina 2G Genome Analyzer and analysed as described previously.^24^ The sequencing data have been submitted to the NCBI Sequence Read Archive and are accessible via accession number PRJNA557177.

### 2.7 Luciferase assay

The *Runx1* + 23 and + 171 elements were inserted downstream of the luciferase gene and the SV40 promoter in the pGL3 vector. pGL3 vector without an enhancer element (and just the SV40 promoter) was used as a negative control. UG26‐1B6 cells were co‐transfected with the test vectors and the pRL‐TK control plasmid and luciferase activity determined as described previously.^25^

## 3 RESULTS AND DISCUSSION

**Figure 1:**
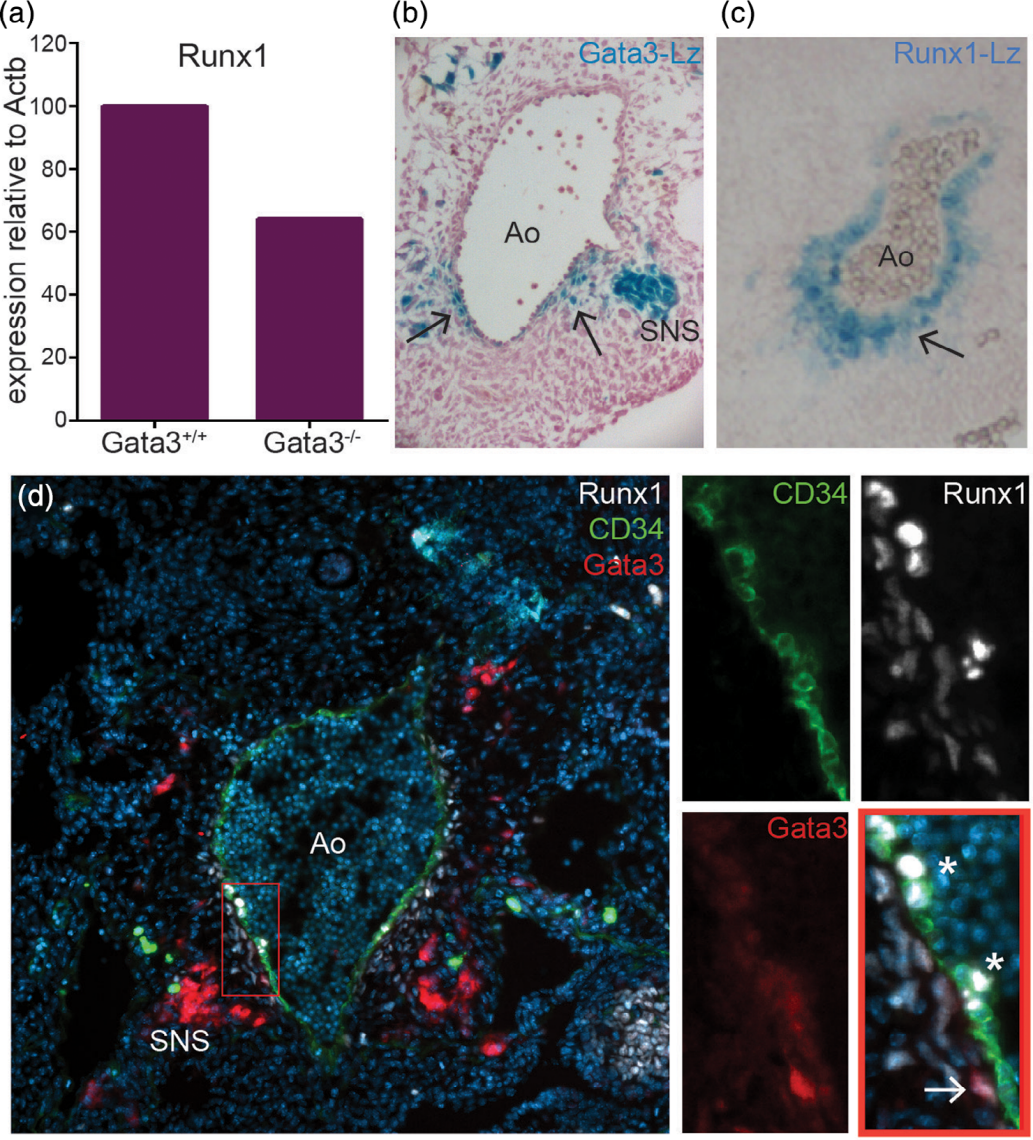
Gata3 and Runx1 co‐localise in the sub‐aortic mesenchyme. (a) *Runx1* expression by qPCR in AGMs from E11 *Gata3* ^*+/+*^ and *Gata3* ^*−/−*^ embryos. (b) *Gata3* expression as detected by X‐gal staining in cryosections from E11 *Gata3* ^*+/lz*^ embryos counterstained with Neutral Red. (c) *Runx1* expression as detected by X‐gal staining in cryosections from E11 *Runx1* ^*+/lz*^ embryos. (d) Immunohistochemistry of a cryosection from a *Gata3* ^*+/GFP*^ E11 embryo with antibodies to GFP (red), Runx1 (white), CD34 (green), and counterstained with DAPI. Smaller panels show a virtual zoom of the region defined by the red box. Asterisks highlight Runx1+ intra‐aortic clusters; arrow points to Runx1 and Gata3 co‐localisation in the sub‐aortic mesenchyme

Our group previously reported a defect in HSC production in the *Gata3* ^*−/−*^ E11 AGM region, which was rescuable by supplying the Gata3‐deficient embryos with an external source of catecholamines.^8^ These results suggested that Gata3 regulates AGM HSC numbers primarily via controlling catecholamine synthesis from the co‐developing SNS. When we repeated the rescue experiments with E10.5 embryos, a time point when mature HSC numbers in the AGM are very low and virtually undetectable in direct transplantations,^5^, ^26^ we were surprised to find repopulation activity specifically in Gata3‐null E10.5 AGMs (Table 1). As expected, no recipients displayed any chimerism with injected E10.5 wild‐type or *Gata3* ^*+/−*^ AGMs, while half of the recipients of E10.5 *Gata3* ^*−/−*^ AGM cells exhibited robust donor contribution (Table 1). These results imply that in catecholamine‐rescued embryos Gata3 deficiency induces a temporal shift in the appearance of HSCs which is SNS‐independent. Such a shift towards an earlier appearance of HSCs in the AGM has previously been observed in Runx1 haploinsufficient embryos.^27^ Intriguingly, we detected a substantial reduction in *Runx1* expression levels (64% of wild‐type) in *Gata3* ^*−/−*^ AGMs (Figure 1a) as reported previously.^8^ This may indicate that Runx1 expression in the AGM is at least partially dependent on Gata3, and that the reduced dose of Runx1 in *Gata3* ^*−/−*^ AGMs may be responsible for the earlier appearance of HSCs.

**Table 1:** HSC activity in AGMs from catecholamine‐treated E10.5 embryos

Genotype	No. of mice repopulated/total transplanted	Percentage chimerism
*Gata3* ^*+/+*^	0/2	0
*Gata3* ^*+/−*^	0/4	0
*Gata3* ^*−/−*^	2/4	60, 13

*Note*: AGMs from E10.5 embryos (35–39 somite pairs) were transplanted at one embryo equivalent directly into irradiated adult recipients and chimerism determined after 4 months. Results are from two independent experiments.

Previous analyses have localised Runx1 expression to intra‐aortic clusters, aortic endothelial cells, and sub‐aortic mesenchyme,^13^, ^21^ while Gata3 is not expressed in intra‐aortic clusters and only in a small subset of aortic endothelial and sub‐aortic mesenchymal cells.^8^ The most substantial overlap between these two transcription factors appears to be in the sub‐aortic mesenchyme (Figure 1b,c), where we detected co‐expression within the same cells (Figure 1d).

**Figure 2:**
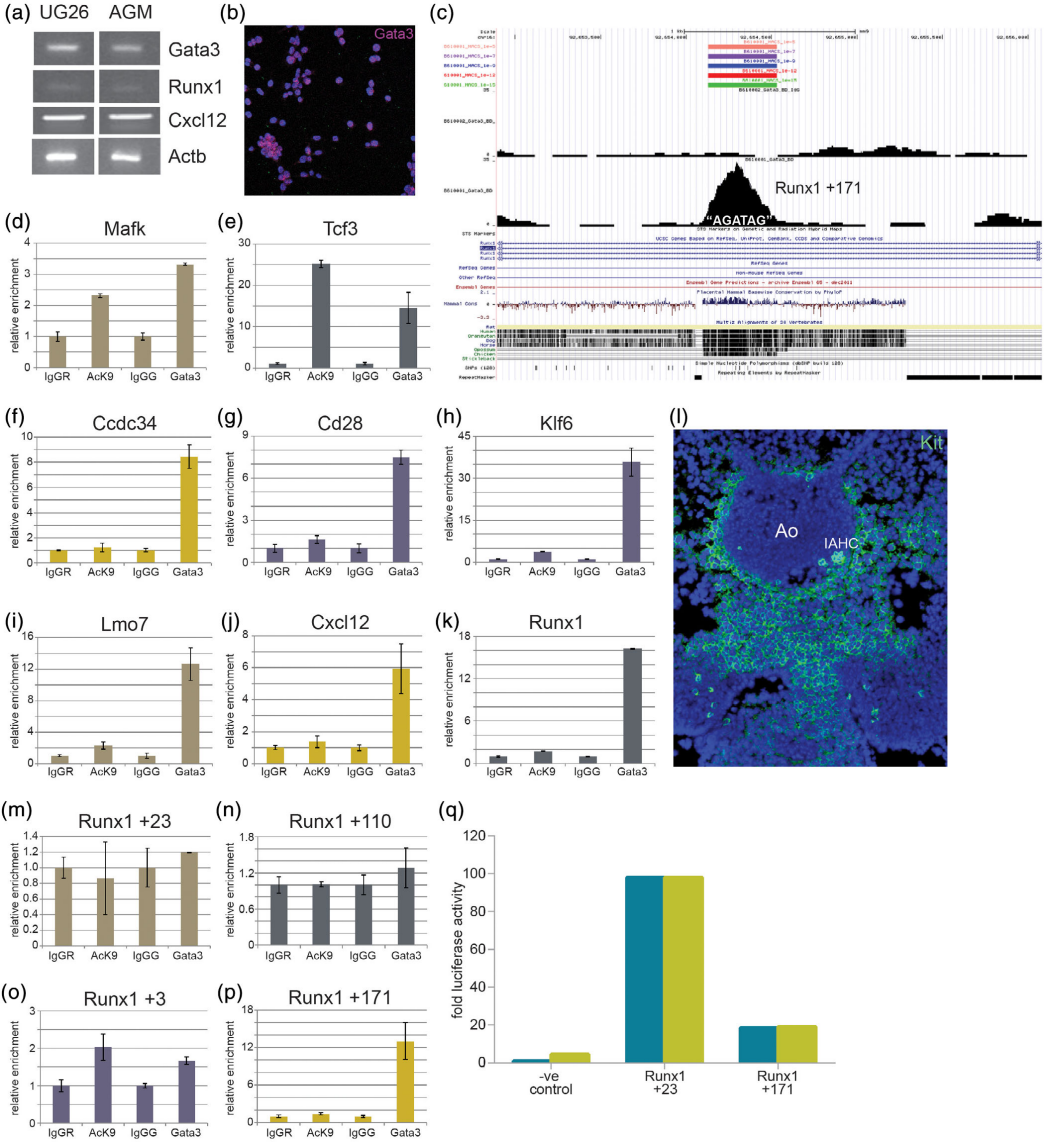
*Runx1* is a direct target gene of Gata3 in AGM‐derived UG26‐1B6 stromal cells. (a) Expression of *Gata3*, *Runx1*, *Cxcl12*, and *Actb* in UG26‐1B6 cells measured by RT‐PCR. (b) UG26‐1B6 cells stained with an antibody to Gata3 (red) and counterstained with DAPI (blue). (c) UCSC Genome browser depiction of the Gata3 binding peak at the +171 enhancer in the *Runx1* locus as measured by ChIP‐Seq. (d–k) ChIP‐qPCR for the indicated gene loci with UG26‐1B6 cell DNA immunoprecipitated with anti‐H3K9Ac (“Ack9”) and anti‐Gata3 antibodies with their respective controls (rabbit IgG [“IgGR”] and goat IgG [“IgGG”]). (l) Immunohistochemistry for Kit (green) on E11 wild‐type embryo cryosections counterstained with DAPI. (m–p) ChIP‐qPCR for the indicated *Runx1* enhancer elements with UG26‐1B6 cell DNA immunoprecipitated with anti‐H3K9Ac (“Ack9”) and anti‐Gata3 antibodies with their respective controls (rabbit IgG [“IgGR”] and goat IgG [“IgGG”]). (q) Replicate Luciferase assays on UG26‐1B6 cells transfected with pGL3 vector without any enhancer elements (negative control) or containing the *Runx1* + 23 and + 171 enhancers. Ao, dorsal aorta; IAHC, intra‐aortic haematopoietic cluster

To determine whether *Runx1* is indeed a direct target of Gata3, we performed ChIP with an antibody to Gata3 followed by massively parallel sequencing (ChIP‐Seq). As this technique requires large cell numbers that would be impossible to obtain from sorted primary sub‐aortic mesenchymal cells, only a subset of which co‐expresses Gata3 and Runx1, we made use of an AGM‐derived stromal cell line, UG26‐1B6, which has been well characterised and used as a model for AGM haematopoiesis‐supportive stroma.^11^, ^13^, ^17^ Most importantly, it expresses both *Gata3* and *Runx1*, as well as *Cxcl12*, a chemokine that was identified as one of the key factors by which niche cells regulate HSC behaviour^28^, ^29^, ^30^ (Figure 2a,b). *Runx1* was indeed identified as one of the most significant Gata3 target genes (Table 2, Figure 2c), and the list of target genes also included *Cxcl12*.

**Table 2:** Most significant Gata3 target genes in UG26‐1B6 AGM‐derived stromal cells as identified by ChIP‐Seq analysis

1.00E‐15	1.00E‐12	1.00E‐09
**Ccdc34**	Adamts14	**Cd28**
Spint1	Ptpru	Atf3
Polr3g1	Hscb	Ccrn41
**Mafk**	Crybb1	Ankrd6
Ano2	Nsmce1	Osbp19
Hpgd	Shank2	Lactb
Lsm4	Tln2	Lep
Ddx39	Rasgrf1	Fam188b
**Tcf3**	Adamts16	**Cxcl12**
Ccdc6	Scd2	Cradd
**Klf6**		Ebf1
Gtf2h2		Xpo4
Nid2		Cdh18
**Lmo7**		Ano6
Ank		Prkd3
**Runx1**		
BC003965		

*Note*: Genes in bold were confirmed by ChIP‐qPCR.

Several of the identified Gata3 targets in Table 2 have also been identified as target genes of multiple key haematopoietic transcription factors,^24^ and we confirmed some of these as top Gata3 targets by ChIP‐qPCR (Figure 2d–k). An antibody against acetylated histone H3 Lysine 9 (Ack9) was also included to confirm accessibility and activity of the identified sites. While Gata3 clearly occupied all of these sites, we could only detect enrichment for Ack9 at the loci for *Mafk* and *Tcf3* (Figure 2d,e). The absence of the Ack9 modification at the other sites (Figure 2f–k) could either mean that Gata3 acts as a repressor of these genes or that it can bind to closed chromatin prior to activation, suggesting that it has pioneer factor activity. Many well‐known reprogramming factors such as Oct4, Sox2 and Klf4 have been described to possess pioneer factor activity.^31^ It is therefore intriguing to note that Gata3 was recently described as a reprogramming factor required for the conversion of mouse fibroblasts into trophoblast stem cells.^32^, ^33^ Whether Gata3 really possesses pioneer factor activity and whether this is important for its function in driving major cell fate decisions during normal development^34^ is an exciting possibility that will require further investigations.

To identify further Gata3 target genes, especially ones that have been linked to haematopoietic processes, we also performed a ChIP‐on‐chip assay, which involved hybridising Gata3‐bound genomic fragments pulled down with the same Gata3 antibody to a customised microarray chip that contained probes of gene loci linked to haematopoiesis (data no shown). Amongst the significant targets were several hits for *Kit*, which in addition to its strong expression in intra‐aortic clusters is also widely expressed in the sub‐aortic mesenchyme (Figure 2l), and *Pdgfra*, a well‐known mesenchymal gene. Most importantly, *Runx1* was once again confirmed as a direct Gata3 target gene.

The genetic elements that regulate the tissue‐specific expression of Runx1 have been well characterised (recently reviewed in^35^). *Runx1* is under the control of two different promoters, which result in the generation of two different isoforms. The distal promoter (P1) is activated in foetal liver and adult haematopoiesis, while the proximal promoter (P2) is active during the early stages of HSC generation from haemogenic endothelial cells.^36^, ^37^ In addition to the two promoters, tissue‐specific expression of Runx1 is also regulated through a number of enhancers, the best studied of which is the +23 enhancer, which targets Runx1 expression to haemogenic endothelial cells and HSPCs.^25^, ^38^, ^39^ To gain insight into the mechanisms through which Gata3 regulates Runx1 expression, we analysed its occupancy on different Runx1 enhancer elements in UG26‐1B6 stromal cells. It showed by far the strongest enrichment at the +171 enhancer with some weak occupancy at the +3 enhancer and no binding at the +23 or + 110 enhancers (Figure 2c,m–p). Luciferase assays confirmed that the +171 enhancer is active in UG26‐1B6 cells, albeit not as strongly as the +23 enhancer (Figure 2q). The activity of the +171 enhancer is comparatively unexplored (compared, for example, with the +23 enhancer), although transient transgenics have shown it to be active in the mouse AGM region (Marella de Bruijn, personal communication). Of note, the human equivalent (+43 kb from the second promoter) of the mouse +171 element was recently shown to contain a DNaseI hypersensitive site (DHS) that is specific to acute myeloid leukaemia samples with internal tandem duplications in the transmembrane domain of FLT3 (FLT3‐ITD).^40^ Furthermore, the human +43 FLT3‐ITD‐specific DHS contains two conserved GATA sites.

Our results have clearly shown that there is an interaction between Gata3 and Runx1 in the embryonic haematopoietic stromal microenvironment and that *Runx1* is a direct target of Gata3 in this context. Whether it is this interaction that can explain the early appearance of HSCs in catecholamine‐rescued *Gata3* ^*−/−*^ AGMs requires further investigation. We attempted to knockdown Gata3 expression in UG26‐1B6 cells in order to assess how this affected Runx1 expression and the ability of these stromal cells to support haematopoietic cells in co‐cultures^13^; however, the knockdown of Gata3 had a negative impact on the viability and/or growth of these cells, which made it impossible to maintain low Gata3 levels during subsequent culture steps (data not shown).

**Figure 3:**
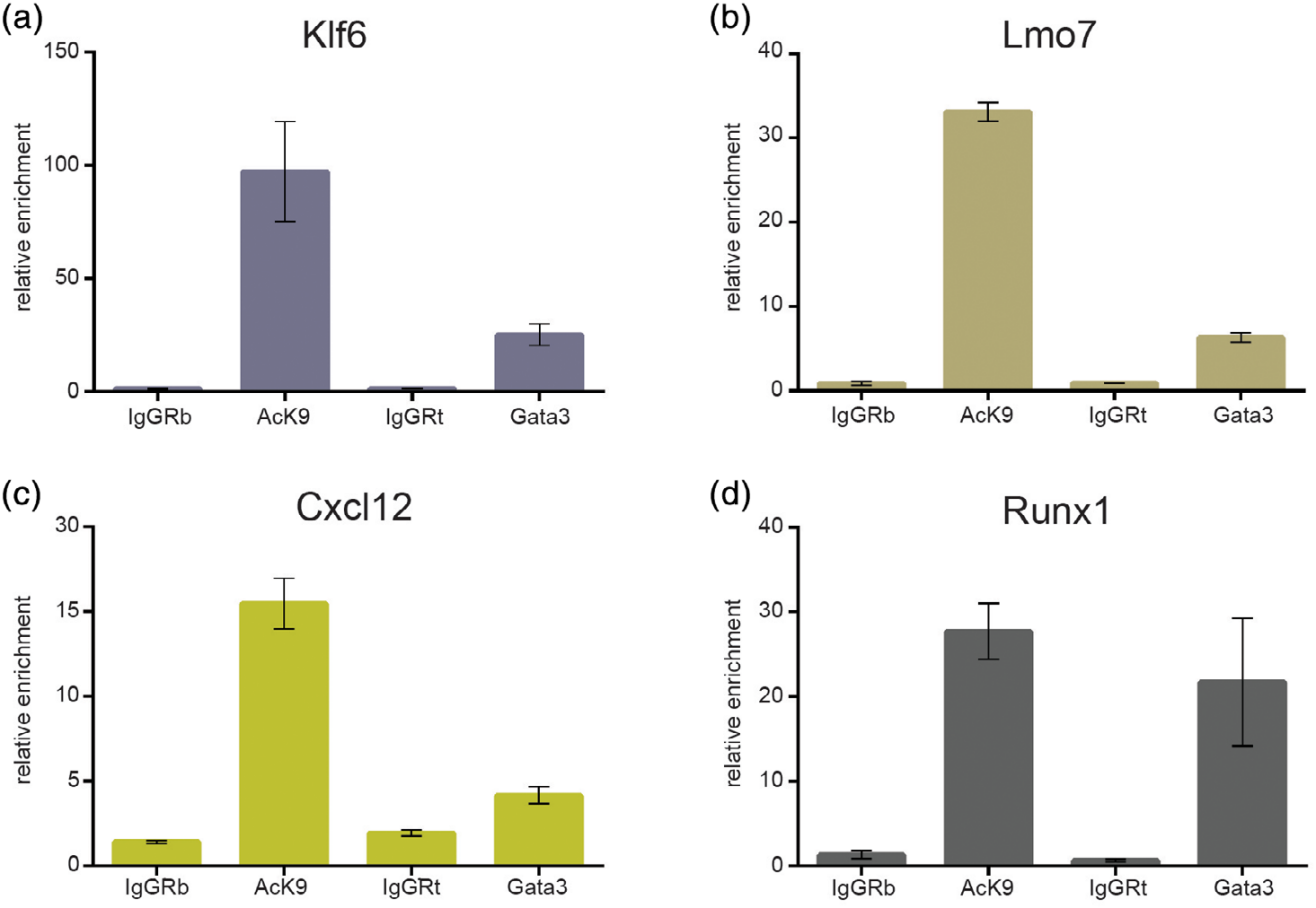
Gata3 binds similar target genes in *Runx1* ^*+/−*^ stromal cells. (a–d) ChIP‐qPCR for the indicated gene loci with KH23 cell DNA immunoprecipitated with anti‐H3K9Ac (“Ack9”) and anti‐Gata3 antibodies with their respective controls (rabbit IgG [“IgGRb”] and rat IgG [“IgGRt”])

It has, however, been previously demonstrated that a half dose of Runx1 results in a more supportive microenvironment through co‐culture experiments with stromal cell lines derived from *Runx1* ^*+/+*^, *Runx1* ^*+/−*^ and *Runx1* ^*−/−*^ AGMs.^13^ We checked whether Gata3 binds to similar targets in the *Runx1* ^*+/−*^ KH23 AGM‐derived cell line by ChIP‐qPCR and confirmed enrichment at the *Klf6*, *Lmo7*, *Cxcl12*, and *Runx1* locus, although these loci appear to be more accessible in this cell line (Figure 3a–d).

In summary, we have demonstrated that *Runx1* is a target gene of Gata3 in the HSPC‐supportive stromal compartment of the AGM region using two different AGM‐derived stromal cell lines as in vitro models. Gata3 appears to bind preferentially to the +171 enhancer element of *Runx1*. Furthermore, the transplantation results as well as the results with the *Runx1* ^*+/−*^ KH23 stromal cell line suggest that the interaction between these two transcription factors may regulate the supportive capacity of the AGM niche, with the exact dose of Runx1 being a critical factor. Interestingly, Gata3 and Runx1 are also both expressed in the haemogenic endothelium of the dorsal aorta.^38^ Whether there is also a direct interplay between these two factors in the haemogenic endothelium and what the nature of this interaction is, is currently under investigation, although unpublished sequencing data from our lab was unable to detect enrichment of *Runx1* expression in Gata3‐positive aortic endothelial cells (Zaidan et al., manuscript in preparation).

## CONFLICT OF INTEREST

The authors declare no potential conflict of interest.
